# A rare case of monomorphic epitheliotropic intestinal T-cell lymphoma, presenting with spontaneous intestinal perforation

**DOI:** 10.1016/j.ijscr.2024.110485

**Published:** 2024-10-20

**Authors:** Chi-Chi Chen, Ching-Ching Chen, Hsiang-Chun Jan, Tzu-Hung Chen, Shao-Jiun Chou, Sheng-Chun Wang

**Affiliations:** Department of surgery, Cardinal Tien hospital, New Taipei City, Taiwan

**Keywords:** Monomorphic epitheliotropic intestinal T-cell lymphoma (MEITL), Enteropathy associated T-cell lymphoma (EATL), Case reports

## Abstract

**Introduction and importance:**

Monomorphic epitheliotropic intestinal T-cell lymphoma (MEITL) is a rare and aggressive T-cell lymphoma that primarily affects the intestine. It has a poor prognosis and high mortality rate. Symptoms at presentation can be non-specific, and imaging studies may show similarities with nonmalignant conditions. The delayed clinical presentation and lack of targeted therapies contribute to the dismal prognosis of MEITL.

**Case presentation:**

We present a case of spontaneous intestinal perforation caused by primary intestinal T-cell lymphoma, emphasizing the importance of early recognition of this uncommon cause of perforation. Identifying it is crucial for prompt surgery and chemotherapy for this rare disease.

**Clinical discussion:**

The most common site of involvement in MEITL is the small intestine, especially the jejunum. The prognosis of MEITL is poor. Early diagnosis of primary intestinal T-cell NHL is challenging due to its rarity and non-specific symptoms. Imaging and endoscopy may show certain features, but a definitive diagnosis relies on biopsy and histopathologic analysis. To date, no efficient therapeutic interventions have been demonstrated for the management of this entity. The standard management strategy consists of induction chemotherapy followed by autologous stem cell transplantation.

**Conclusion:**

This case report highlights that spontaneous perforation with peritonitis could be a potential presenting sign of MEITL. The diagnosis of MEITL is mainly based on histopathologic examination, so an accurate diagnosis necessitates clinical knowledge and thorough biopsy with immunohistochemistry and molecular testing.

## Highlights

• MEITL is a rare and aggressive T-cell lymphoma that primarily affects the intestine.
• It has a poor prognosis and high mortality rate.
• The delayed clinical presentation and lack of targeted therapies contribute to the dismal prognosis of MEITL.
• The diagnosis is mainly based on histopathologic examination, so an accurate diagnosis necessitates clinical knowledge and thorough biopsy with immunohistochemistry and molecular testing.
• Treatment options such as chemotherapy, surgery, or radiotherapy have not been proven to improve the outcome.
• More research is needed to understand the molecular pathogenesis of MEITL and develop effective treatment strategies.

## 1 Introduction

MEITL is a relatively rare disease previously known as type II of Enteropathy associated T-cell lymphoma, accounting for approximately 5 % of gastrointestinal lymphomas and less than 1 % of all non-Hodgkin lymphomas. MEITL has been independently identified as a subtype of lymphoma by the World Health Organization (WHO) since 2017. It is caused by proliferation of intraepithelial lymphocytes [1]. The initial presenting symptoms of primary T-cell NHL of the GIT are non-specific, with abdominal pain being the most common chief complaint. Perforation is a well-known complication of intestinal NHL due to chemotherapy; however, perforation as the initial presentation of primary GI tract lymphoma is extremely rare [2]. MEITL has been independently identified as a subtype of lymphoma by the World Health Organization (WHO) since 2017. MEITL currently lacks a standard treatment regimen, and usually follows the cyclophosphamide, doxorubicin, vincristine, prednisone, etoposide (CHOPE) regimen for T-cell lymphoma. However, the curative effect of current chemotherapy is not good. Hematopoietic stem cell transplantation may improve outcomes in patients with MEITL [1]. Here we present a rare case of primary intestinal T-cell lymphoma in a patient who presented with severe acute abdominal pain and imaging studies suggestive of intestinal tumor with possible bowel perforation.

## 2 Case report

**Fig. 1:**
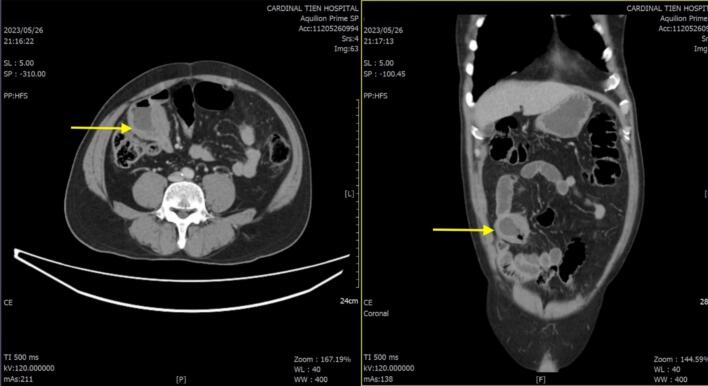
Computed tomogram (CT) of the abdomen with contrast revealing focal dilatation and annular wall thickening of the distal ileu.

**Fig. 2:**
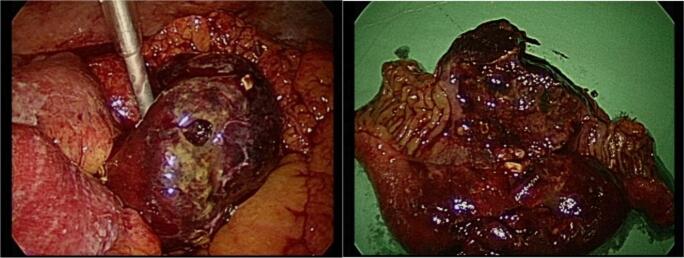
Nearly circumferential infiltrative ulcerative mass (2b) with a perforation hole (2a).

A 58-year-old man with a history of hypertension and coronary artery disease presented to the emergency department (ED) complaining of sudden onset of right lower abdominal pain for one day. He had been experiencing abdominal discomfort for a week. Laboratory tests showed elevated levels of C-reactive protein and neutrophil leukocytosis. A computed tomogram (CT) of the abdomen with contrast revealed focal dilatation and annular wall thickening of the distal ileum (Fig. 1) without pneumoperitoneum. No extraintestinal lesions were found on the CT scan. Under general anaesthesia, he received laparoscopy with three trochars and segmental resection and side to side anastomosis of the distal jejunum, finally drained with two drainage (Jackson-Pratt drains). The surgical specimen (jejunum) showed a segment of a nearly circumferential infiltrative ulcerative mass with a perforation hole (Fig. 2a,b).

Pathologic examination revealed prominent epitheliotropism, transmural infiltrate, and necrosis with CD3+, CD4+, and CD56+. The final diagnosis was MEITL (monomorphic epitheliotropic intestinal T-cell lymphoma). Bowel movement started on post op day 1 and he started his oral diet on post op day 7. Then the patient was ultimately referred to hematology for the adjuvant chemotherapy: initiation of cyclophosphamide, doxorubicin, vincristine, and prednisone (CHOP). He received one cycle of chemotherapy with the first-line CHOP regimen (cyclophosphamide, doxorubicin, vincristine and prednisone) and lost follow up later.

## 3 Discussion

Intestinal T-cell lymphoma is a rare tumor that makes up 0.25 % of all malignant lymphomas and less than 5 % of primary malignant lymphomas of the digestive tract. It is caused by the malignant proliferation of intraepithelial lymphocytes. Intestinal T-cell lymphomas are classified into different subtypes, including enteropathy-associated T-cell lymphoma (EATL), monomorphic epitheliotropic intestinal TCL (MEITL), indolent T-cell lymphoproliferative disorder of the gastrointestinal tract, and intestinal T-cell lymphoma, NOS. EATL is the most common subtype, predominantly found in Europe and North America [3].

MEITL occurs at a wide range of ages with a median age of 58 years and with a male to female ratio of 2:1 [4]. The most common site of involvement in MEITL is the small intestine, especially the jejunum [5,6,7]. The stomach, duodenum, and large intestine may also be affected, with reported rates of 2.4–12.0 %, 31 %, and 8.3–23 %, respectively [7,8,9]. Symptoms include abdominal pain, diarrhea, weight loss, and GI hemorrhage [8,10]. Since MEITL is not associated with celiac disease, there is typically no history of malabsorption, and it is often detected after acute abdominal symptoms caused by intestinal obstruction or perforation. The prognosis of MEITL is poor with a previously reported median overall survival of only 7 months [11] and 1-year overall survival is only 36 % [4].

Early diagnosis of primary intestinal T-cell NHL is challenging due to its rarity and non-specific symptoms [12]. Imaging and endoscopy may show certain features, but a definitive diagnosis relies on biopsy and histopathologic analysis [13].

No abnormality found under CT cannot completely exclude MEITL, so it plays a sufficient and unnecessary role in the diagnosis of MEITL [14]. Endoscopically, MEITL appears as a single or multiple masses, deep ulcers, superficial ulcers, or relatively normal findings. Mass formation and superficial ulceration are most frequently seen, in approximately 40 % of cases [7].

Tumor cells in MEITL are typically CD3^+^, CD5^−^, CD7^+^, CD4^−^, TIA-1^+^, granzyme B^+^, perforin^+^, and CD103^+^. Most MEITL cells are CD8^+^ and CD56^+^, unlike EATL. Aberrant CD20 expression has been reported in 11–24 % of cases, underscoring the need for caution in diagnosis. CD30 is generally negative [6,10]. Megakaryocyte-associated tyrosine kinase (MATK) is positive in 87 % of tumor cells in MEITL, and the extent of MATK expression has been reported to be useful for differentiating MEITL from EATL [6,15].

To date, no efficient therapeutic interventions have been demonstrated for the management of this entity. The standard management strategy consists of induction chemotherapy followed by autologous stem cell transplantation. Chemotherapy regimens used include anthracycline-based regimens, such as CHOP, IVE/MTX, and l-Asparaginase-based regimens. However, regardless of the scheme used, the differences are not usually very significant with regard to survival. A study conducted by Yi J et al. showed that there was no significant difference regarding the complete remission rate between CHOP and non-CHOP regimens (37 % vs 71 %, *p* = 0.095) [16] Other studies have shown anthracycline-based regimen have a lower complete remission rate than l-asparaginase-base regimens. [17]

## 4 Conclusion

This case report highlights that spontaneous perforation with peritonitis as a potential presenting sign of primary intestinal T-cell NHL. The diagnosis of NHL strictly relies on histopathologic analysis; thus, in patients with spontaneous perforation of unknown etiology, it is crucial to obtain intraoperative biopsies of the perforation and perform an endoscopic evaluation of intestinal mucosa after surgical recovery to evaluate for suspicious lesions. Given the rarity of this subtype of lymphoma, early recognition of this uncommon cause of perforation is essential to ensure expedited hematology referral and initiation of appropriate treatment.

## Ethical approval

Include in IRB CTH-112-3-7-006.

Ethical approval for this study (CTH-112-3-7-006) was provided by the Ethical Committee of Cardinal Tien Hospital, New Taipei City, Taiwan on 13 October 2023.

## Funding

Personal.

## Author contribution

Correspondence to: Ching-Ching Chen

Writing the paper: Chi-Chi Chen

Guidance of all: Sheng-Chun Wang, Hsiang-Chun Jan, Shaw-Jiun Chou, Tzu-Hung Chen.

## Guarantor

Chi-Chi Chen.

## Consent

Written informed consent was obtained from the patient for publication and any accompanying images. A copy of the written consent is available for review by the Editor-in-Chief of this journal on request.

## Registration of research studies

No registry

## Conflict of interest statement

The authors declare that they have no conflict of interest regarding the publication of this paper.
